# A Narrative Review of Postoperative Anticoagulation Therapy for Congenital Cardiac Disease

**DOI:** 10.3389/fsurg.2022.907782

**Published:** 2022-06-14

**Authors:** Alexander A. Boucher, Julia A. Heneghan, Subin Jang, Kaitlyn A. Spillane, Aaron M. Abarbanell, Marie E. Steiner, Andrew D. Meyer

**Affiliations:** ^1^ Division of Pediatric Hematology and Oncology, Department of Pediatrics, University of Minnesota Masonic Children’s Hospital, Minneapolis, MN, United States; ^2^ Division of Critical Care, Department of Pediatrics, University of Minnesota Masonic Children’s Hospital, Minneapolis, MN, United States; ^3^ Division of Pediatric Cardiac Surgery, Department of Surgery, University of Minnesota Masonic Children’s Hospital, Minneapolis, MN, United States; ^4^ Division of Critical Care, Department of Pediatrics, Long School of Medicine, University of Texas Health Science Center, San Antonio, TX, United States; ^5^ Division of Congenital Cardiac Surgery, Department of Cardiothoracic Surgery, Long School of Medicine, University of Texas Health Science Center, San Antonio, TX, United States

**Keywords:** Blood coagulation, congenital heart disease, aspirin, warfarin, anticoagulants, pediatrics

## Abstract

Congenital heart disease encompasses a range of cardiac birth defects. Some defects require early and complex surgical intervention and post-operative thromboprophylaxis primarily for valve, conduit, and shunt patency. Antiplatelet and anticoagulant management strategies vary considerably and may or may not align with recognized consensus practice guidelines. In addition, newer anticoagulant agents are being increasingly used in children, but these medications are not addressed in most consensus statements.

This narrative review evaluated the literature from 2011 through 2021 on the topic of postoperative thromboprophylaxis after congenital heart disease operations. The search was focused on the descriptions and results of pediatric studies for replacement and/or repair of heart valves, shunts, conduits, and other congenital heart disease operations. Wide variability in practice exists and, as was true a decade ago, few randomized controlled trials have been conducted. Aspirin, warfarin, and perioperative heparin remain the most commonly used agents with varying dosing, duration, and monitoring strategies, making comparisons difficult. Only recently have data on direct oral anticoagulants been published in children, suggesting evolving paradigms of care. Our findings highlight the need for more research to strengthen the evidence for standardized thromboprophylaxis strategies.

## Introduction

Congenital heart disease (CHD) has one of the highest birth prevalence rates of any congenital anomaly (1, 2). Surgical advancements have significantly increased the lifespan of children with CHD; however, postoperative complications of bleeding and thrombosis remain a significant cause of morbidity and mortality (3). CHD is also independently associated with non-cardiac hospital-acquired venous thromboembolism in the multi-institutional Children’s Hospital-Acquired Thrombosis registry (4). Despite these recognized risks, methods of immediate and/or long-term anticoagulation after congenital heart surgery remain variable and controversial. This is likely due to a combination of few prospective, multicenter studies along with the fact that anticoagulation management often depends on physician specialty, surgeon expertise, and medical center experience (5). This variability may increase the rate of complications which may be avoided with improved evidence-based standardization across medical centers.

International collaborations have developed clinical practice guidelines for anticoagulation strategies, though these are frequently based mostly on low-evidence expert consensus rather than strong bases of evidence (5–8). The 9^th^ Edition of the American College of Chest Physicians’ “Antithrombotic Therapy in Neonates and Children” guidelines, often simply referred to as “the *CHEST* guidelines,” are among the most commonly referenced pediatric anticoagulation guidelines for specialists in many different areas. Yet, the most current update is now a decade old and does not cover all of the currently available anticoagulation and antiplatelet agents, with a scope of review from 1999 to early 2010 (9). The last guideline from the American Heart Association was in 2013 (“Prevention and Treatment of Thrombosis in Pediatric and Congenital Heart Disease”) (5). Since that time, new therapies and strategies have emerged, including direct thrombin inhibitors (DTI), and have gained favor over traditional therapies such as unfractionated heparin. Furthermore, direct oral anticoagulants (DOAC) have increasingly demonstrated safe and effective results in variety of pediatric studies (10–14). In this narrative review, we comprehensively examined the available literature on anticoagulation and antiplatelet strategies for postoperative pediatric CHD care since 2011. Our goal was to determine the extent to which these strategies have been standardized and where advancements have been made.

## Materials and Methods

### Data sources and Study Eligibility

In our first search on July 31, 2020, English-language publications from January 2011 to July 2020 were identified using the MEDLINE, Embase, and Cochrane databases. All searches included the search terms: anticoagulants, antithrombins, platelet aggregation inhibitors, heart defects (congenital), heart valve diseases, heart valve prosthesis implantation, heart valve prosthesis, bioprosthetic heart valve, mechanical heart valve, modified Blalock-Taussig-Thomas (mBTT) shunt, central shunt, Norwood, Norwood-Sano, bilateral cavopulmonary, Glenn, Fontan, adolescent, infant, child, young adult. Studies that involved DOACs, DTIs, or antiplatelet therapies besides aspirin were included. The authors then assessed for the following inclusion criteria: (1) original research studies, (2) pediatric studies with CHD populations, and (3) surgical interventions. The authors also repeated the literature search with same search terms on January 10, 2022, to identify additional papers which may have been published since the first search.

### Exclusion Criteria

Initial search criteria excluded all studies published prior to January 2011 that included the following: adult age or middle age. Further exclusion criteria were studies with animals or exclusively adult populations, those for which only an abstract was available, and non-English-language articles. After the initial data collection, the authors excluded all duplicates, case reports including <10 patients, review manuscripts, background literature, opinion pieces, manuscripts without evidence of anticoagulation being reported, clinical trial announcements without results, and conference abstracts.

### Database Construction and Data Definitions

The resultant manuscripts were divided into the following categories: valves, shunts, conduits, and “other procedures” [e.g., transposition of the great arteries (TGA) repair, septal defect repair]. Some manuscripts fit multiple categories depending on the design of the study or the data reported on multiple CHD defects. Any manuscript that overlapped categories is listed in each of the respective tables. Many manuscripts included pediatric and adult data. Since pediatric data were the primary focus of this review, we excluded adult data if possible; if they could not be separated and the primary data were not available, we described the overall findings with a notation that adult data was included. Study information including publication year, funding, study location, population demographics, time frame of study (i.e., early postoperative period versus later follow up), procedure/lesion(s), and bleeding or thrombotic complications were independently extracted into a custom REDCap database by at least two authors (15). Discrepancies were resolved by full author consensus. “Major” versus “minor” bleeding reports are described based on the criteria of the respective papers. For the purposes of this review, “short-term” postoperative refers to the first week after an operation including bridging and “long-term” refers to data reported after the first postoperative week. The term “anticoagulation” refers to anticoagulants and antiplatelet agents unless otherwise specified. Enoxaparin dosing was presumed to be subcutaneous unless specifically noted in the studies.

## Results

### Study Selection

**Figure 1:**
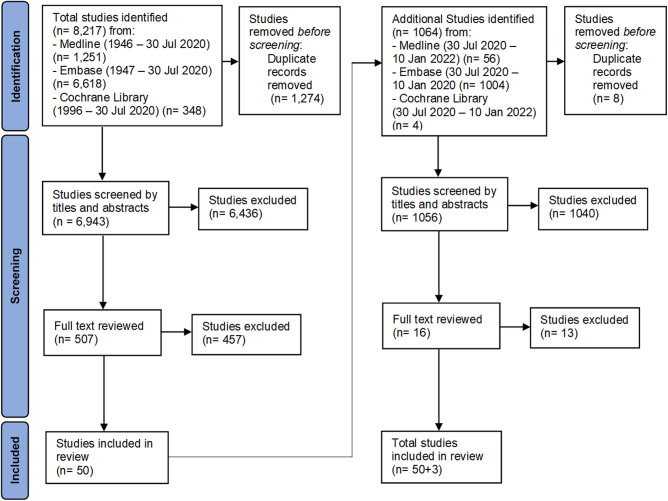
The flow chart shows the details of the inclusion and exclusion of manuscripts for review.

The flow diagram of study selection to final data extraction for analysis is in Figure 1. The first literature search yielded >8,000 records. Duplicates (1,274) were removed, leaving a total of 6,943 abstract records in the first review. Using Rayyan, a web-based systematic review software, authors AAB and SJ reviewed all available titles and abstracts and excluded publications based on the previously listed criteria, reducing the reviewable manuscripts to 507 in total (16). All authors reviewed these selected manuscripts reducing the final count to 50 manuscripts. In the second literature search in January 2022, a total of 1064 additional papers published in the interim were found. After applying the same evaluation criteria in the first search, 3 additional papers were included, for a total of 53 manuscripts.

## Valves

### Study Characteristics

Sixteen manuscripts related to valve surgeries were reviewed (Table 1). Studies included clinical experience for both mechanical and bioprosthetic valves. Nine manuscripts included multiple valves within the same data set [e.g., mitral valve (MV) and aortic valve (AV) repairs] and included either mechanical or bioprosthetic categories. Two included both mechanical and bioprosthetic valves in the same manuscript. Most were retrospective, primarily single site, chart reviews, though one prospective observational trial focused on implementation of a heparin protocol with a primary aim to describe monitoring assay outcomes and another evaluated whether aspirin efficacy testing predicts thrombotic events.

**Table 1:** Valve study descriptions.

Author	Year	N	Age Range^a^	Procedure	Study Type	Anticoagulation^b^	Complications (hemorrhagic and/or TE)	Comments
Alsoufi	2011	307	Mean 11.4 years (SD 5.6)	MV, AV, TV (Mech 75%, BP 25%)	R	Heparin (1.5 – 2× baseline aPTT) Warfarin (INR 2.5–3.5) Rare ASA	3% valve/cardiac TE (4.2% overall TE) 6% bleeding (10% associated with anticoagulation)	None
Bliss	2011	38	1.1–17.2 years	AV, MV	R	Warfarin (INR 2–3.5) Enoxaparin only used when bridging to warfarin	7 mild/moderate bleeds	Focus was point-of-care warfarin monitoring at home
Dos	2011	22	14–50 years	PV (Mech)	R	Warfarin (INR 2.5–3.5) Antiplatelet use poorly defined	8 TE 2 moderate bleeds	TE only occurred in 2 patients. TE linked to overt right heart failure and occurred late after surgery
Mahle^c^	2011	86	0.3–20.8 years	AV, MV, TV (BP)	R	Warfarin	3 major bleeds (1.3% major bleeds/patient year on warfarin)	Long-term warfarin better managed with computer algorithm. Risk of warfarin-related admission associated with BP valves
Sfyridis^e^	2011	34	0.2–46 years	PV (BP)	R	Warfarin (INR 1.5–2 for 3 months)	1 conduit valve TE on anticoagulation 4 valve TE without anticoagulation	None
Wong	2011	25	10.4 years (Mean)	AV, MV, TV (All BP)	R	Warfarin	3 major bleeds (12%)	None
Brown	2012	97	2 weeks– 18 years	MV (83% Mech, 17% BP)	R	Warfarin (INR 2.5–3.5 for initial 3–4 months, then INR 2–3) ASA 80 mg	10% “systemic emboli” 6% valve TE 3% major bleeds requiring transfusion	Extended review of MV replacement survival and complications. Few Anticoagulation details
Lowry^c^	2012	45	0.2–45 years	AV, MV, TV (44 Mech, 1 BP)	R	Warfarin (58% bridged with heparin/ enoxaparin)	9.8% TE	None
Sim	2012	19	3 months– 16 years	MV (Mech)	R	Warfarin (INR 2.5–3)	2 minor bleeds	None
Emani^c,d^	2014	26	<18 years	28% “Complex valves”	P	Heparin ASA (20.25, 40.5, 81 mg/day) – median dose 6.5 mg/kg/day	7.5% TE (more common in non-responders – 1.2% versus 60%)	ASA unresponsiveness using VerifyNow-ASA™ Thrombotic events not described by specific procedures
Gupta	2015	24	3 months– 20.3 years	AV (BP)	R	Warfarin immediately postoperatively (INR 2.5), then ASA or clopidogrel for 6–12 weeks	None	None
Moon	2015	18	4 days– 7 months	MV (BP)	R	Heparin (2–2.5× baseline aPTT) Warfarin (INR 2–3) Dipyridamole 4 mg/kg/day	3 (16.6%) with ICH (2 deaths)	None
Murray^c^	2015	240	5 months– 55 years	MV, AV (BP)	R	88% Warfarin 28% Enoxaparin	None	Focus on anticoagulation program
Hwang	2016	89	2.6–48 years	PV (BP)	R	ASA	None	≥12 months ASA no better than <12 months after PV replacement
Nair^c,d,e^	2018	203	Median 0.33 years (IQR 0.03–2.37)	AV, MV, PV+ other procedures	P	Heparin infusion protocol ASA Clopidogrel Tirofiban Warfarin Enoxaparin	Risk ratio for TE 0.76 pre-intervention vs post (*P* = 0.55) “Clinically relevant” bleeding events: 4.14 events/100 PD pre-intervention vs 1.62 events/100 PD post-intervention No difference in major bleeds	Protocol decreased bleeding but no change in TE
Oladunjoye^c,d^	2018	202	Median 0.4 years (IQR 0.1–2.3)	AV, MV, PV, TV (6.4% of cases)	P	Heparin infusion protocol per Nair et al. Median dose 24 U/kg/hour (IQR, 20—32 U/kg/hour) 66.3% ASA	9.4% TE (1.68 events/100 PD) Major bleeds: 14.2% (3.02 events/ 100 PD)	aPTT > 150 s more predictive than Xa for bleeding

*aPTT, activated partial thromboplastin time; ASA, aspirin/acetylsalicylic acid; AV, aortic valve; BP, bioprosthetic valve; ICH, intracranial hemorrhage; INR, international normalized ratio; IQR, interquartile range; Mech, mechanical valve; MV, mitral valve; P, prospective observation/intervention study, PD, patient days; PV, pulmonary valve; R, retrospective study; SD, standard deviation; TE, thromboembolism; TV, tricuspid valve; U, units.*

^a^ *Ages represent ranges unless otherwise specified.*

^b^ *Anticoagulation includes antiplatelet therapy as well as monitoring strategy and targets if described.*

^c^ *C* *Study also included in Conduit Table (Table 3).*

^d^ *Study also included in Shunt Table (Table 2).*

^e^ *Study also included in Other Procedures Table (Table 4).*

Valve manuscripts included between 17—307 participants, though 44% of manuscripts included adult patients and did not clearly delineate pediatric-only findings. The age range of pediatric-only studies was 4 days – 18 years. For the entire group, AV and MV repairs were the most commonly reported procedures (nine studies each, 40.1%) with some manuscripts including both.

### Short-Term Management

Short-term anticoagulation was reported or inferred in nine studies, the majority involving mechanical valves. Heparin was primarily used early in the postoperative course, with dosing ranging from a constant 10 units/kg/hour to therapeutic dose titration. Heparin was then bridged to warfarin by 24–48 h following surgery in six of these eight studies and enoxaparin was rarely employed. One manuscript described bridging to warfarin with therapeutic enoxaparin or heparin in 58% of cases but in this study, the time to reaching the goal international normalized ratio (INR) was quite variable (1—13 days) (17). Antiplatelet therapy was infrequently used in the early postoperative course, and no DOAC or DTI use was reported.

### Long-Term Management

Warfarin was the primary long-term therapy of choice for valve operations (14/16 manuscripts). Antiplatelet therapy was used in only 50% of studies. Aspirin was used as monotherapy in bioprosthetic pulmonary valve (PV) replacement in a study aiming to determine the optimal duration of therapy (18). Those researchers found that short courses (approximately 6 months) of aspirin as primary prevention were non-inferior to using aspirin for ≥12 months. Another manuscript reported a transition from early warfarin to aspirin (or clopidogrel for some patients in the study) for 6–12 weeks (19). Dipyridamole 4 mg/kg/day was used in a separate study along with warfarin for newborns and infants who underwent bioprosthetic MV replacement (20). Antiplatelet agents were used in five other manuscripts, though details were scant (21–25). DOAC use was rare—a single patient received dabigatran among all the studies (22).

### Monitoring

Heparin therapeutic dosing targets ranged from an activated partial thromboplastin time (aPTT) of 1.5–2 to 2–2.5 times the baseline for MV, AV, and tricuspid valve replacements. Studies reported an INR goal of 2.5–3.5 was used for mechanical valves, while 2–3 was used in most other situations. Brown et al. reported using an initial warfarin INR goal of 2.5–3.5 for bioprosthetic and mechanical MV replacement, though the goal INR was lowered to 2–3 after –4 months (24). One study reported use of a lower INR goal (1.5–2) at that institution for 3 months after PV replacement, though its efficacy was not compared to standard INR goals (26). In one study of 27 patients with complex valve repairs, laboratory-based resistance using VerifyNow-Aspirin™ was seen more commonly with lower dose aspirin with 28.6% resistance using 20.25 mg (27). This laboratory-based aspirin resistance was overcome with higher aspirin dosing, as the researchers found a reduction to 6% resistance using 40.5 mg and 5.3% resistance using 81 mg. The authors did note that the population receiving the lowest dose of aspirin was both the smallest group (*n* = 7) and the youngest subset of patients, with the latter putting them at the highest developmental risk for aspirin unresponsiveness. None of the other studies reported testing for aspirin efficacy.

### Complications

Major bleeding complications ranged from 2.0–16.6%. Specifics on bleeding episodes were inconsistently reported, though in the study with the highest percentage (16.6%), the events were characterized as chronic subdural hematomas diagnosed 1–7 months after MV replacement, all while on warfarin (20). Thromboembolic incidence ranged from 0–36.3% across studies (18, 19, 21, 28–32). Dos et al. reported a high rate of thromboembolism (36.3%), although these events occurred in only 9% of patients, as eight thrombotic events occurred between two patients, many occurring months to years valve surgery (21). Two studies reported thrombosis incidence in events per patient days. In one, there were 1.68 thromboembolic events/100 patient days (25), while in the other, a study evaluating complications before and after implementation of a heparin standardization protocol, saw a reduction in thromboembolic incidence from 1.48 events/patient days pre-standardization to 1.12 events/patient days afterwards (28). Overall, intracardiac or valve thromboses were more frequently reported thromboembolic complications as compared to systemic venous thromboembolism.

### Summary

For valve operations, nearly all were retrospective, single site studies. Warfarin was the most common long-term thromboprophylaxis agent used regardless of the type of valve operation, though short-term bridging was inconsistent. Antiplatelet therapy was documented in 50% of the studies, most of which included multiple types of valve operations. Bleeding and thrombotic outcomes were rarely reported and inconsistently defined, though most thromboses were intracardiac or involved the valves themselves.

## Shunts

### Study Characteristics

Twenty-three manuscripts included data on mBTT shunts with or without Norwood procedure, central shunts, or Norwood/Sano procedures (Table 2). Of these, one was a randomized control trial (RCT) (33), while the remainder were retrospective cohort studies focused on patient outcomes. Subjects typically underwent procedures in the first week of life, consistent with the typical age range for these procedures. Sample size ranged from 10—932 patients.

**Table 2:** Shunt study descriptions.

Author	Year	N	Age Range^a^	Procedure	Study Type	Anticoagulation^b^	Complications (hemorrhagic and/or TE)	Comments
Januszewska	2011	236	2–82 days	Norwood with RV-PA shunt	R	Heparin 5–10 U/kg/hour “if no bleeding” ASA 2–5 mg/kg/day until second stage	2.5% shunt occlusion	Shunt-related complications after Norwood
Ohman	2012	28	3–35 days	mBTT, central, or Sano shunt	P	ASA 5 mg/kg/day Warfarin (*N* = 1) ASA+ Warfarin (*N* = 2)	29% Shunt narrowing	SpO2 monitoring at home
Manlhiot^e^	2012	195	3–74 days	All three stages of SV palliation (Stage I *N* = 145)	CSS	66% Enoxaparin (anti-Xa 0.5–1 U/mL) 30% no prophylaxis 2% ASA+ enoxaparin 2% ASA	49% Arterial/cardiac thromboses 9% Stroke/PE 51% Venous thrombosis	Thrombotic complications across all three stages of SV palliation; Multiple TE in some patients
Tzanetos^e^	2012	16	Mean 4.6 days (Norwood)	All three stages of SV palliation (Stage I Norwood *N* = 5)	P	Heparin 10 U/kg/hour ASA starting postoperative day 1–2	31% thrombus on prospective imaging (3 of 5 had Norwood done previously)	TE in SV patients associated with lower ATIII levels, higher tPA antigen levels, longer bypass time, cardiac dysfunction on preoperative echo
Guzzetta	2013	207	Mean 20 days	mBTT	R	Heparin 10 U/kg/hour ASA 40 mg/day	6.8% Shunt occlusion	In-hospital shunt thrombosis in mBTT
Romlin	2013	14	3–100 days	mBTT/Sano, Norwood	P	Heparin 250 U/kg/day ASA 3–5 mg/kg/day	None reported	Observational study of impedance aggregometry
Wessel	2013	906	0–92 days	Systemic-PA shunts	RCT	Clopidogrel 0.2 mg/kg/day versus placebo plus usual care 87.9% ASA	Shunt thrombosis: 5.8% of clopidogrel, 4.8% placebo 3.8% major bleeds	Bleeding was most commonly gastrointestinal
Bao	2014	110	1–228 months	Central shunt	R	Heparin 0.25 mg/kg infusion every 6 hours x3 days Aspirin 3–5 mg/kg/day	1.8% Shunt thrombosis	None
Emani^d,e^	2014	95	<18 years	15% Stage I/ mBTT	P	Heparin ASA (20.25, 40.5, 81 mg/d) – median dose 6.5 mg/kg/day	7.5% TE (more common in non-responders – 1.2% versus 60%)	ASA unresponsiveness using VerifyNow-ASA™ Thrombotic events not described by specific procedures
Horer	2014	13	Not reported	Systemic-PA shunt	P	Heparin 5000 U/m^2^/day ASA 3–5 mg/kg/day	7.7% VTE (no shunt thrombosis	Histopathologic study of explanted shunts
Mir	2015	20	4–75 days	Norwood/Sano, mBTT	P	ASA 20 mg/day; if resistance +, dose increased to 40 mg/day	no bleeding or thrombotic complications	80% ASA resistance in SV patients
Kucuk	2016	44	1 day–20 months	mBTT	R	Heparin ASA 3–5 mg/kg/day	9.1% shunt thrombosis	Risk for adverse outcomes after mBTT
Anderson	2017	80	Not reported	Systemic-PA shunt +/− Norwood/DKS	R	ASA 20 mg/day	15% shunt thrombosis; (6.3% within 24 h)	Hematocrit was risk for shunt thrombosis
Chittithavorn	2017	85	1–123 days	mBTT	R	Heparin 10 u/kg/h “if no bleeding” ASA 2–5 mg/kg/day	14% Shunt thrombosis	<3 mg was risk factor for in-hospital shunt thrombosis after mBTT
Ramachandran	2017	932	Median 5 days (IQR 4–8)	Stage I and II patients – 932 stage I only here (56% Norwood/RV-PA, 41% Norwood/ mBTT, 3% central/other)	R (MCR)	93.8% aspirin (87% solidary), 4% no anticoagulants	0.3% shunt thrombosis 0.2% with stroke (clopidogrel or enoxaparin only)	Variation in antithrombotic therapy in SV patients across sites
Truong^e^	2017	24	2–352 days	Norwood (/BT), BT/central, cavopulmonary	P	ASA 3–5 mg/kg/day	8.3% TE (No shunt thrombosis) 20.8% major bleeds	Suboptimal AA inhibition
Ambarsari	2018	51	3–83 days	Heparin-bonded systemic-PA shunts 72.5% univentricular	R	ASA 3 mg/kg until shunt takedown	9.8% Shunt thrombosis	
Nair^d,e,f^	2018	792	Median 0.33 years (IQR 0.03–2.37)	Variety of procedures, shunts not divided out	P	Heparin infusion protocol ASA Clopidogrel Tirofiban Warfarin Enoxaparin	Risk ratio for TE 0.76 pre-intervention versus post (*P* = 0.55) “Clinically relevant” bleeding events: 4.14 events/100 PD pre-intervention versus 1.62 events /100 PD post-intervention No difference in major bleeds	Protocol decreased bleeding but no change in TE
Oladunjoye^d,e^	2018	202	Median 0.4 years (IQR 0.1–2.3)	9.4% after stage I palliation + other procedures	P	Heparin infusion protocol per Nair et al. Median dose 24 U/kg/hour (IQR, 20–32 U/kg/hour) 66.3% ASA	9.4% TE (1.68 events/100 PD) Major bleeds: 14.2% (3.02 events/ 100 PD)	aPTT > 150 s more predictive than Xa for bleeding
Saini	2019	68	Mean 0.31 months	mBTT	R	ASA dosing not described (<7 mg/kg/day low, ≥8 mg/kg/day high)	16.2% Shunt thrombosis (15% in standard dose, 18% in high-dose, statistically similar)	Pre/post evaluation of different ASA doses
Leijser^f^	2019	118	11–12 days	30% SV 70% TGA	P	Heparin 28 U/kg/hour (anti-Xa 0.35–0.75 U/mL) Enoxaparin 1.75 mg/kg every 12 h (anti-Xa 0.5–1 U/mL) ASA used 5–10 mg/kg/day (not universally used but few details)	*AIS:* 10.8% TGA 17% SV *SDH:* 24% TGA 23% SV *IVH:* 6% TGA 2.8% SV *Parenchymal stroke:* 37% SV	Focused on stroke prophylaxis (30% had AIS, 56% had TE preoperatively Increased risk postoperative stroke on anticoagulation in SV but not TGA New white matter changes seen more often in newborns with anticoagulation + AP (80%) vs anticoagulation only (29%)
Okamoto	2020	41	5–68 days	systemic-PA shunt	R	Heparin (target aPTT 45– 50 seconds) ASA 3–5 mg/kg/day)	1 death from shunt obstruction	Functional SV heart with extracardiac TAPVR
Erdem	2021	103^c^	mean 56 ± SD 51 months	18.4% systemic-PA shunt + mixed procedures	P	ASA 3–5 mg/kg/day up to 100 mg/day Six months treatment if repaired with synthetic patches, transcatheter devices, or stents	36.9% ASA resistance; this group was more likely to have had a history of TE (34.2% versus 9.2%)	Platelet aggregation as measured by AggreGuide A-100

*aPTT, activated partial thromboplastin time; AA, arachidonic acid; AIS, acute ischemic stroke; AP, antiplatelet therapy; ASA, aspirin/acetylsalicylic acid; ATIII, antithrombin III; CSS, cross-sectional study; DKS, Damus–Kaye–Stansel procedure; INR, international normalized ratio; IQR, interquartile range; IVH, intraventricular hemorrhage; mBTT, modified Blalock-Taussig-Thomas shunt; MCR, multicenter registry; P, prospective observation/intervention study, PA, pulmonary artery; PD, patient days; R, retrospective study; RV, right ventricle; SD, standard deviation, SDH, subdural hemorrhage; SV, single ventricle; TAPVR, Total anomalous pulmonary venous return; TE, thromboembolism; TGA, transposition of the great arteries; tPa, tissue plasminogen activator; U, unit.*

^a^ *Ages represent ranges unless otherwise specified.*

^b^ *Anticoagulation includes antiplatelet therapy as well as monitoring strategy and targets if described.*

^c^ *Only 19 were aorticopulmonary arterial shunts.*

^d^ *Study also included in Valve Table (Table 1).*

^e^ *Study also included in Conduit Table (Table 3).*

^f^ *Study also included in Other Procedures Table (Table 4).*

### Short-Term Management

Heparin infusions were the most used initial anticoagulation strategy once postoperative hemostasis was ensured. Dosing ranged from 10–28 units/kg/hour when reported, though one study used 5000 units/m^2^ (34). Patients were often transitioned to aspirin within three days of surgery, usually linked to timing of enteral feeds, though one study included patients who transitioned from heparin to enoxaparin (35). Aspirin doses primarily fell between 3–5 mg/kg/day (or 20–40.5 mg/day), consistent with the recommended pediatric dosing for pediatric modified BTT shunt palliation (1–5 mg/kg/day) provided in the *CHEST* guidelines (9). Leijser et al. compared variations of secondary stroke prophylaxis between two Canadian pediatric surgical centers that included infants with BTT, where one site used a continuous heparin infusion of 28 units/kg/hour for thromboprophylaxis while the other did not routinely use anticoagulation other than aspirin 5–10 mg/kg/day per provider discretion (35).

### Long-Term Management

The most common long-term anticoagulation strategy for shunt procedures was aspirin monotherapy, typically dosed at 2—5 mg/kg/day (20—40.5 mg/day). The dose that was chosen early in the postoperative course was then stably maintained throughout treatment. One RCT compared clopidogrel use plus standard-of-care aspirin versus aspirin plus placebo and did not show additional benefit in dual therapy for the prevention of thromboembolism or mortality (33). One Canadian study used enoxaparin as primary prophylactic anticoagulation (anti-Xa goal 0.5—1.0 units/mL), although its efficacy was not compared to other strategies (36). This study also reported the highest proportion of patients (30%) who did not undergo anticoagulation at the time of discharge after initial palliation. A review of the National Pediatric Cardiology Quality Improvement Collaborative registry demonstrated 87% of patients who had undergone initial palliation for hypoplastic left heart syndrome and related variants were discharged on aspirin monotherapy, 6.8% on a multidrug regimen, 1.7% on enoxaparin alone. The other 4% received no anticoagulant therapy (37). While several studies examining shunt procedures included patients with Sano shunts, anticoagulation management did not differ from other shunt procedures.

### Monitoring

Several studies describe aspirin efficacy monitoring in the short-term postoperative time window. A variety of testing platforms to determine “aspirin resistance” were used, including VerifyNow-Aspirin™ (10.5% incidence of “aspirin resistance”) (27), thromboelastography with platelet mapping (TEG-PM™, 13% incidence across three time points in one study, 80% in a separate study) (38, 39), urine thromboxane levels (100% incidence) (39), or impedance aggregometry (7—36% incidence, depending on time point) (40). The high but variable rates of aspirin resistance are consistent with previous publications highlighting the discordance among aspirin resistance testing methods (41–43). Of note, most of these studies primarily reported laboratory-based aspirin resistance, which often do not have validated pediatric cutoffs, nor are the results necessarily equivalent to clinical breakthrough thromboembolism on aspirin, also known as “high on-treatment platelet reactivity.” Whether changes were made to antithrombotic therapy in response to laboratory assessment of aspirin efficacy is not well described in these manuscripts. One study described an adjustment of aspirin dosing from 20 to 40 mg/day if laboratory-based aspirin resistance was noted (no thromboses were reported) (39). However, reaching therapeutic thresholds proved difficult, likely due to a combination of clinical factors, adult testing cutoffs, and developmental hemostasis. Monitoring of other routine coagulation assays were not consistently associated with decreased rates of postoperative shunt thrombosis (44, 45).

### Complications

Bleeding and thrombosis complications were inconsistently reported in our review of shunt manuscripts. Major bleeding complications reported in nine manuscripts included central nervous system, gastrointestinal, thoracic/chest tube, pulmonary, and other poorly defined bleeding sites, with rates ranging from 0.5—12.5%. Thrombotic complications were reported in 17 manuscripts (range 0—30%), with shunt thrombosis being most common. In Leijser et al., in which they focused on stroke incidence, the authors documented an increased incidence of postoperative stroke, primarily acute ischemic stroke, in those who received anticoagulation for BTT as compared to those without anticoagulation. The authors of this study concluded that this was evidence of anticoagulation’s limited benefit in reducing neurologic morbidity in these cases (35). Some study authors attempted to identify factors associated with bleeding and thrombotic complications, yet were significantly limited by small patient populations, single-site studies, and the inclusion of patients without shunts. Shunt occlusion was associated with pulmonary atresia/ventricular septal defect (VSD) with or without major aortopulmonary collateral arteries and small pulmonary artery size (45). Heparin-coated polytetrafluoroethylene shunts did not result in histopathologic differences compared to uncoated shunts, nor did their use eliminate the risk for shunt thrombosis (34, 46). One single-center study of 68 patients compared “low” (<7 mg/kg/day) and “high” (≥8 mg/kg/day) empiric dosing of aspirin and found no difference in shunt thrombosis rates (47). One study reported shunt complications and interstage mortality when considering a variety of diagnoses, surgical interventions, and shunt types (48). The small number of patients included in this single study limit any conclusions regarding the association of shunt type with outcomes.

### Summary

Following shunt procedures, aspirin was the most employed thromboembolism prophylaxis. The incidence of aspirin resistance (or high on-treatment platelet reactivity) using conventional platelet inhibition thresholds, which have not been validated in children, was high but widely variable. Furthermore, increasing the aspirin dose after laboratory-based aspirin resistance was noted did not consistently mitigate thromboembolic risk. Adding weight-based clopidogrel to conventional therapy also did not reduce all-cause or shunt-related morbidity in neonates or infants with CHD palliated with a systemic-to-pulmonary artery shunt (33). Both bleeding and thromboembolic outcomes were common in this high-risk population, even in the face of seemingly adequate platelet function inhibition.

## Conduits

### Study Characteristics

Twenty-three studies were included for review, including some with more than one conduit procedure reported (Table 3). Five manuscripts mentioned Glenn procedure (e.g., uni- and bi-directional, cavopulmonary connection, or Kawashima) and seventeen mentioned Fontan procedure (e.g., extracardiac, lateral tunnel, and aortopulmonary). Two manuscripts were focused on the same RCT for long-term Fontan thromboprophylaxis in a cohort who underwent primarily extracardiac conduits (49, 50). One new RCT (UNIVERSE Study) published in 2021 compared the DOAC rivaroxaban with aspirin for twelve months in Fontan patients (51). The remainder of the studies included retrospective, prospective, or cross-sectional cohorts and one provider survey. Five papers described either anticoagulation protocols or anticoagulation monitoring of a number of CHD lesions (25, 28, 29, 52, 53). The multicenter studies included two large RCTs, one published by Monagle et al., in 2011 and the other by McCrindle et al., in 2021 (50, 51), one survey study of 16 pediatric cardiology and cardiothoracic network centers in the United Kingdom (54), and one multicenter cohort study on anticoagulation in Fontans from New Zealand and Australia. Sample size ranged from 16—490 patients, though not all were limited to conduits.

**Table 3:** Conduits study descriptions.

Author	Year	N	Age Range^a^	Procedure	Study Type	Anticoagulation^b^	Complications (hemorrhagic and/or TE)	Comments
Mahle	2011	86	0.4–20.8 years	4.6% SV	R	Warfarin	1.3% per patient year	None
Monagle & McCrindle^c^	2011 2013	111	Mean ASA arm 4.6 years (SD 2.3) Mean Warfarin arm 5.1 years (SD 3.4)	EC 86% 85%	RCT	50% ASA 50% Warfarin	TE: 21% ASA 24% Warfarin Major bleeds: 2% ASA 2% Warfarin Minor bleeds: 14% ASA 33% Warfarin	ASA non-inferior to warfarin for thromboprophylaxis at 2 years
Lowry^e^	2012	11	Median 14.3 years (0.2–44.8)	Fontan	R	Warfarin	Bleeding incidence: 5%	Data specific to Fontan characteristics not available
Manlhiot^d,f^	2012	139	Median 28 months (IQR 13–44)	82% AP 16% Hybrid 12% BDG	CSS	54% None 35% Enoxaparin 6% ASA 4% Enoxaparin + ASA 1% Warfarin	TE: 16% inpatient, 12% after discharge No bleeds reported	
Manlhiot^d,f^	2012	162	Mean 3.4 years (SD 1.4)	22% LT 76% EC 2% Other	CSS	77% Warfarin 17% ASA 6% None	TE: 16% (50% inpatient) Bleeds: 1%	None
Tzanetos^f^	2012	4	Mean 154 months (SD 26.5)	Unspecified Glenn	R	Heparin 10 U/kg/hour + ASA	25% TE (fatal IVC thrombosis)	Small number of patients with Glenn procedures
Crone	2013	47	2–25 years	Variety of procedures	R	87% Warfarin 4.7% ASA	None reported	Study outcome based around INR monitoring program
Potter	2013	210	4.8–15.2 years	60% AP 39% LT 2% EC	R	50% None 25% Warfarin 25% ASA	TE: 27% without thromboprophylaxis 13% on warfarin 9.8% on ASA No bleeds reported	None
Emani^f^	2014	36	Mean age by dose: 3 weeks (20.25 mg) 5.3 months (40.5 mg) 3.25 years (81 mg)	15 Fontan 8 Baffle 7 Arterial Switch 6 BDG	P	ASA 7.3% 20.25 mg 52.6% 40.5 mg 40% 81 mg	TE rates by dose: 2.6% 20.25 mg 6% 40.25 mg 5.3% 81 mg No bleeds	Very few received 20.25 mg (*N* = 7) and they were all <1 month old
Ohuchi	2014	412	Mean 4.7 years (SD 5.3)	60% EC 25% LT 15% AP	R	27% ASA 15% Warfarin + ASA 15% Warfarin transitioned to ASA 15% None	24% TE 7% Bleeds	None
Thomas	2014	32	Mean 2.3 years	97% LT	R	ASA + warfarin	No TE 3% bleeds	None
Murray^e^	2015	240	5 months–55 years	20% Fontan	R	88% Warfarin 28% Enoxaparin	None	Focus on anticoagulation program
Galantowicz	2016	Pre-protocol *N* = 64 Post-protocol *N* = 55	Pre-protocol median 5.6 months (SD 1.4) Post-protocol median 5.9 months (SD 1.9)	Stage 2 Cavopulmonary connection or BDG	CC	Heparin at 24 h Enoxaparin for 6 weeks, then ASA	TE: PA thromboses most common Pre-protocol 11% Post-protocol 0% Bleeds: Pre-protocol 16% Post-protocol 5%	Evaluation of alternative surgical strategy for HLHS
Patregnani	2016	20	Median 33 months	Fontan	P	ASA	20% TE No bleeding	Study evaluating HOTPR after Fontan (50%) using multiple methods
Faircloth	2017	89	Median 8.3 years (IQR 6.8–11.4)	79% EC 21% LT	R	63% Warfarin + ASA 15% ASA 7.5% Warfarin 21% “Multiple therapy changes”	TE 9.8%	None
Truong^f^	2017	10	2–352 days	6 Glenn (25%) 2 BDG (8%) 2 Kawashima (8%)	P	ASA 3–5 mg/kg/day	8.3% TE (No shunt thrombosis) 20.8% major bleeds	Suboptimal AA inhibition
Nair^e,f,g^	2018	203	Median 0.33 years (IQR 0.03–2.37)	Mixed procedures	P	Heparin infusion protocol ASA Clopidogrel Tirofiban Warfarin Enoxaparin	Risk ratio for TE 0.76 pre-intervention versus post (*P* = 0.55) “Clinically relevant” bleeding events: 4.14 events/100 PD pre-intervention versus 1.62 events /100 PD post-intervention No difference in major bleeds	Protocol decreased bleeding but no change in TE
Oladunjoye^e,f^	2018	202	Median 0.4 years (IQR 0.1–2.3)	21.3% SV 5.9% Baffle	P	Heparin infusion protocol per Nair et al. Median dose 24 U/kg/hour (IQR, 20—32 U/kg/hour) 66.3% ASA	9.4% TE (1.68 events/100 PD) Major bleeds: 14.2% (3.02 events/ 100 PD)	aPTT > 150 s more predictive than Xa for bleeding
Al-Jazairi	2019	431	Mean 2.4 years (SD 8.4)	71% LT 25% EC 1.6% AP	R	89.3% Warfarin 5.1% ASA 5.7% None	TE: 9.9% >1 year (17/1,000 patient-years at median 13.5 years after surgery) 3.9% Major bleeds 15.3% Minor bleeds	Evaluation of late TE after Fontan out to 20 years. Low rates of late (>1 year) TE; no difference between ASA or warfarin
Singh	2020	N/A	N/A	92% EC 8% LT	CSS	58% Warfarin 42% ASA	Not Reported	Survey of provider variability in management strategies
Ankola	2021	192	2.8–3.9 years	56% EC 44% LT	R	54% ASA 27% Warfarin 7% None 4% Heparin	10% TE No bleeds reported	None
Attard	2021	490	16.9–27.6 years	49% EC 31% LT 21% AP	R	66% Warfarin 34% ASA	Epistaxis: 47% warfarin, 23.5% ASA Stroke: *Symptomatic:* 5% Warfarin, 7% ASA *Asymptomatic:* 44% Warfarin, 34% ASA	Long-term post-Fontan study Most strokes after Fontan were asymptomatic, microhemorrhage and white matter changes were common, regardless of thromboprophylaxis
McCrindle	2021	100	Mean 4.1 years (SD 1.7)	Fontan	RCT	67% Rivaroxaban 33% ASA	Major bleed: 6% Rivaroxaban 9% ASA Trivial bleed: 33% Rivaroxaban 35% ASA	First RCT with DOAC

*aPTT, activated partial thromboplastin time; AA, arachidonic acid; AP, aortopulmonary conduit; ASA, aspirin/acetylsalicylic acid; BDG, bidirectional Glenn; CC, case/control study; CSS, cross-sectional study; DOAC, direct oral anticoagulant; EC, extracardiac conduit; HLHS, hypoplastic left heart syndrome; INR, international normalized ratio; IQR, interquartile range; IVC, inferior vena cava; LT, lateral tunnel conduit; N/A, not available; P, prospective observation/intervention study, PD, patient days; R, retrospective study; RCT, randomized controlled trial; SD, standard deviation; SV, single ventricle; TE, thromboembolism; U, units.*

^a^ *Ages represent ranges unless otherwise specified.*

^b^ *Anticoagulation includes antiplatelet therapy as well as monitoring strategy and targets if described.*

^c^ *The manuscripts by Monagle et al. and McCrindle et al. evaluate different aspects of the same study.*

^d^ *The manuscript by Manlhiot et al. described two separate cross-sectional studies so they were separated for this table.*

^e^ *Study also included in Valve Table (Table 1).*

^f^ *Study also included in Shunt Table (Table 2).*

^g^ *Study also included in Other Procedures Table (Table 4).*

### Short-Term Management

Ten manuscripts describe early postoperative prophylaxis. Three manuscripts reported heparin infusions along with aspirin, two including heparin infusions only, two included warfarin monotherapy, one included warfarin with enoxaparin bridging, and two compared warfarin versus aspirin (although these reviewed the same RCT). Details on management were minimal except in the RCT of Fontan patients by Monagle et al., where participants assigned to warfarin received a flat-rate continuous heparin infusion of 10–20 units/kg/hour until first oral intake (54). Patients assigned to aspirin in this trial had no heparin phase and no monitoring. Three studies reported use of a flat-rate continuous heparin infusion of 10 units/kg/hour prior transition to aspirin or warfarin, though both Emani et al. and Lowry et al. mention that a large percentage of participants in their studies did not receive heparin (29.5% and 50%, respectively) (17, 27, 55). Emani et al. examined low (20.25 mg), middle (40.5 mg) and high dose (81 mg) aspirin escalation by patient age in a mixed population, all started early after surgery (27). Nair et al. described the most detailed regimen for immediate postoperative prophylaxis, although this was not limited to patients with conduits (28). Based on the perioperative clinical status and bleeding risks as determined by the intensivist and the attending surgeon, a tiered algorithm with a heparin dose titration matrix was used, including defining an initial anticoagulation strategy [therapeutic, intermediate (15 units/kg/hour), or line/prophylactic heparin (10 units/kg/h, only for patients <10 kg)], time window for initial anticoagulation, and bridging strategy.

Four papers mention using enoxaparin in the early postoperative period, usually as a bridge to warfarin (17, 36, 56, 57). Manlhiot et al. detailed their use of enoxaparin prophylaxis starting with a dose of 1.5 mg/kg/day (age ≤2 months) or 1 mg/kg/day (non-infants), representing 50% of the expected treatment dose, with the dose titrated to reach an anti-Xa level of 0.5–1.0 units/mL (36). The two studies that focused on anticoagulation protocols recommended starting warfarin at 0.1 mg/kg/dose (max 10 mg) with titration over 2–7 days to reach goal INR (28, 52). Both of papers were from a single institution that stated that the indication and timing for initial anticoagulation, duration, and bridge strategy were decided by the primary surgeon. Specifically, Nair et al. describes three options for initial heparin anticoagulation to be therapeutic, intermediate, and line heparin (28). The therapeutic heparin protocol targeted an aPTT <100 s and anti-Xa between 0.35–0.7 units/mL, starting six hours postoperatively. The intermediate heparin option was 15 units/kg/hour (maximum rate 750 units/hour) with daily labs to ensure the patient was not supratherapeutic. The line heparin was only for patients <10 kg and to be kept at 10 units/kg/hour with no lab monitoring. In the Monagle et al. RCT for Fontan, a loading dose of 0.1 mg/kg of warfarin was used with titration to a goal INR of 2–3 over the next week, bridging with initial heparin 10–20 units/kg/hour. No manuscripts described clopidogrel, DOAC, or DTI use in the early postoperative period. Finally, Ankola et al. reported that most (93%) of their 192 Fontan patients were started on some form of postoperative thromboprophylaxis, with 54% of those treated started on aspirin, 27% on warfarin, 7% on enoxaparin, and 4% on heparin, starting on median postoperative day 4 (IQR 2–6) (57).

### Long-Term Management

Like the first stage palliation procedures (shunts), the most prescribed thromboprophylactic agent was aspirin. Manlhiot et al. reported an anticoagulation strategy for all three stages (36). In this study involving 139 Glenn-like second-stage procedures, 54% received no thromboprophylaxis, 35% received enoxaparin only, 6% received aspirin only, 4% received enoxaparin and aspirin, and only 1% received warfarin monotherapy (36). In contrast, for the 162 post-operative Fontan patients in the study, 77% were prescribed warfarin, 17% received aspirin monotherapy, and 6% received no thromboprophylaxis. Aspirin was non-inferior to long-term warfarin thromboprophylaxis after two years in one large RCT of 111 Fontan patients, although both were deemed suboptimal in terms of thrombosis incidence and the dosing was infrequently reported (50). One manuscript did describe DOAC use in conduits. This was the UNIVERSE study, published in 2021, which was an RCT comparing rivaroxaban to aspirin as thromboprophylaxis (51). This study was an RCT comparing rivaroxaban to aspirin after Fontan as thromboprophylaxis in 100 participants randomized in a 2:1 ratio. Lastly, Singh et al. analyzed surveys from 12 of 16 pediatric network centers in the United Kingdom documenting a wide variation of thromboprophylaxis strategies with warfarin being the most commonly utilized first-line agent (58%) (54).

### Monitoring

Similar to the previous section on shunts, several manuscripts covering conduits described various aspirin efficacy monitoring methods. The time point after surgery also played a role in laboratory-based aspirin resistance using TEG-PM™ (38). In this study, 38% had >50% arachidonic acid inhibition (representing appropriate antiplatelet activity) after the third aspirin dose, 60% at the first postoperative clinic visit, but then only 26% 2–8 months after surgery.

Oladunjoye et al. compared aPTT to anti-Xa levels in heparinized pediatric patients after CHD surgery, finding the former was more predictive of bleeding (25). Of note, this study was not limited to conduits. The risk of bleeding increased significantly when aPTT exceeded 150 seconds (odds ratio 1.11, 95% confidence interval 0.89–1.29). For INR monitoring, compliance with target INR improved if the physician used a multidisciplinary team (52) or a computer-directed program (29). In one study analyzing factors that affect hospital length of stay after Fontan including time to reach therapeutic INR levels, 34% of children never achieved a target INR while in the hospital (53). Interestingly, they also found that Fontan patients required a smaller loading dose to achieve a therapeutic INR (0.09 mg/kg; 95% confidence interval 0.08–0.11) compared to a historic study that reported twice this dose was necessary to achieve therapeutic INR (0.18 mg/kg) (58).

### Complications

Despite the frequent use of anticoagulation, bleeding complications were rarely reported. Only the RCT by Monagle et al. documented significant minor bleeding with warfarin compared to aspirin (33% versus 13%, *P* < 0.03) (53). The manuscripts describing anticoagulation protocols or coagulation monitoring did not report bleeding or thrombotic outcomes in detail. Attard et al. in 2021 documented a variety of different complications associated with thromboprophylaxis in a cohort of patients in New Zealand and Australia (59). Specifically, in 100 patients after Fontan operations in this study, warfarin use had a higher incidence of epistaxis compared to aspirin (47% compared to 23.5%). Eighty-four Fontan patients documented no significant difference between aspirin or warfarin in the incidence of clinical or radiographic cerebrovascular injury. However, another subset of 120 patients with Fontans in Attard et al. receiving warfarin had significantly reduced bone mineral density compared to patients on aspirin. In the UNIVERSE study, clinically relevant minor bleeding occurred 6% in participants on rivaroxaban compared to 9% of participants on aspirin (51). One major bleeding event (epistaxis requiring transfusion) occurred in the rivaroxaban group with none in the aspirin group. The investigators concluded that the drugs had similar safety profiles and there was no significant difference in bleeding and thrombotic outcomes between the two interventions.

For studies that did describe thromboembolic complications, they were identified following Glenn procedures with the incidences ranging from 8.3% – 28.6% (27, 36, 38, 55, 56). Thromboembolism rates were similar for Fontan patients (16%–24%) (36, 60–62). No difference in major thrombotic or bleeding outcomes using either aspirin or warfarin was found in the RCT comparing the two medications after Fontan operations (50). In one study of 192 patients after Fontan completion, the overall incidence of ultrasound-documented thrombosis was 10% within the first 30 postoperative days, primarily intracardiac in location (57). Only 7% (*N* = 14) in the Ankola et al*.* study received no thromboprophylaxis; of these, 79% developed a Fontan circuit thrombosis (57). These authors further documented that no children starting thromboprophylaxis before postoperative day 2 developed thromboses and the percentage of patients developing thromboses increased steadily with each day that thromboprophylaxis was delayed. Finally, they found that Fontan thrombosis was associated with longer lengths of stay, mechanical ventilation days, and chest tube duration. Potter et al. reported that thromboprophylaxis was protective against thromboembolism development over a 20-year postoperative period compared to no treatment (52% versus 86% incidence, respectively) (62). In the one RCT involving rivaroxaban, the authors reported one participant on rivaroxaban had a pulmonary embolism (2% overall event rate) while for aspirin, one participant had ischemic stroke and 2 had venous thrombosis (9% overall event rate) (51).

### Summary

Aspirin and warfarin are the most reported thromboprophylaxis agents used for conduits, though the type of surgery heavily influenced the medication used and whether combination therapy was employed. This was the only group of procedures where DOAC efficacy was evaluated as well. Aspirin monitoring has been trialed with varying results, as seen with other procedures. Some study demonstrate that dose-adjusted aspirin is associated with reduced thrombotic incidence, and there may be a role for multidisciplinary or computer-based algorithms for warfarin dosing and monitoring. In the RCT by Monagle et al., warfarin had higher bleeding risk for postoperative CHD than aspirin. The risk of conduit thrombosis is high in both the early and late postoperative periods—conduit-related thromboses may occur decades later—and thromboembolic increases with delays in prophylactic anticoagulation initiation. The role and risk/benefit balance of DOACs is uncertain based on limited data, yet the UNIVERSE study was an important first step to expand the therapeutic options for pediatric conduit thromboprophylaxis.

## Other Procedures: Septal Defects, Tetralogy of Fallot, and Transposition of the Great Arteries

### Study Characteristics

Given the paucity of stand-alone available manuscripts, we chose to group the four studies with significant percentages of septal defects, Tetralogy of Fallot (ToF), and transposition of the great arteries (TGA) repair together into this section (Table 4). Atrial septal defects and VSD have historically not been managed with anticoagulation. In reviewing the literature, most septal defect repairs receiving anticoagulation were part of larger, more complex cardiac surgeries. One manuscript primarily focused on septal defect repair including patent ductus arteriosus (63), one described ToF outcomes (64), and another described TGA repair along with patients who have single ventricle physiology (35). Three other publications also documented patients with these CHD lesions, yet they were mixed in with other lesions and have been described in other sections (26, 28, 65). This group includes one prospective multisite observational cohort (35), one prospective study of protocol implementation (28), and three retrospective cohort studies (26, 63, 65). The number of participants in these studies ranged from 11–209 and included ages ranging from 11 days—56 years. It should be noted that most of these studies involved adult patients; only two studies limited the age range to those ≤18 years old (35, 64). The studies that included adults did not provide enough detail to solely characterize pediatric data, so full cohort data is described.

**Table 4:** Study descriptions for septal defects, tetralogy of Fallot, and transposition of the great arteries.

Author	Year	N	Age Range^a^	Procedure	Study Type	Anticoagulation^b^	Complications (hemorrhagic and/or TE)	Comments
Guo	2011	209	3–56 years	ASD VSD PDA	R	ASA 3–5 mg/kg/day started at 24 h, given 3–6 months	2 hemothoraces	Primary outcome focused on OPOTTMIS operation outcome
Sfyridis^c^	2011	34	0.2–46 years	41% TOF (BP valve)	R	Warfarin (INR 1.5–2 for 3 months)	1 valve TE on warfarin, 4 TE without warfarin No bleeding reported	None
Hashmi	2017	11	5 mo– 19 years	Various procedures	R	Warfarin (44%) ASA (13%)	6/7 with anticoagulation had postoperative bleeding (minor) 1 had bleeding intraoperatively	Focused on acquired von Willebrand Syndrome in cardiopulmonary disorders
Nair^c,d,e^	2018	203	Median 0.33 years (IQR 0.03–2.37)	ASD VSD TOF + others	P	Heparin infusion protocol ASA Clopidogrel Tirofiban Warfarin Enoxaparin	Risk ratio for TE 0.76 pre-intervention versus post (*P* = 0.55) “Clinically relevant” bleeding events: 4.14 events/100 PD pre-intervention versus 1.62 events /100 PD post-intervention No difference in major bleeds	Protocol decreased bleeding but no change in TE
Leijser^d^	2019	118	11–12 days	70% TGA 30% SV	P	Heparin 28 U/kg/hour (anti-Xa 0.35—0.75 U/mL) Enoxaparin 1.75 mg/kg every 12 h (anti-Xa 0.5–1 U/mL) ASA used 5–10 mg/kg/day (not universally used but few details)	*AIS:* 10.8% TGA 17% SV *SDH:* 24% TGA 23% SV *IVH:* 6% TGA 2.8% SV *Parenchymal stroke:* 37% SV	Focused on stroke prophylaxis (30% had AIS, 56% had TE preoperatively Increased risk postoperative stroke on anticoagulation in SV but not TGA New white matter changes seen more often in newborns with anticoagulation + AP (80%) vs anticoagulation only (29%)

*AIS, acute ischemic sAP, antiplatelet therapy; ASA, aspirin/acetylsalicylic acid; ASD, atrial septal defect; IQR, interquartile range; OPOTTMIS, off-pump occlusion of trans-thoracic minimal invasive surgery; PDA, patent ductus arteriosus; P, prospective observational study; R, retrospective study; SV, single ventricle physiology; TGA, transposition of the great arteries; TOF, Tetralogy of Fallot; U, units; VSD, ventricular septal defect*

^a^ *Ages represent ranges unless otherwise specified.*

^b^ *Anticoagulation includes antiplatelet therapy as well as monitoring strategy and targets if described.*

^c^ *Study also included in Valve Table (Table 1).*

^d^ *Study also included in Shunt Table (Table 2).*

^e^ *Study also included in Conduits Table (Table 3).*

### Short-Term Management

Continuous heparin infusion was reportedly used in 40% of these studies, though dosing varied when reported. In Leijser et al., the authors compared two institutions with different protocols around secondary stroke prophylaxis after TGA repair and mBTT in infants (ages 11–12 days), some of whom received anticoagulation therapy while others did not (35). One site started a heparin infusion of 28 units/kg/hour without a loading dose, titrated to a goal heparin anti-Xa level of 0.35–0.75 units/mL as standard practice for TGA while the other site gave a 75 units/kg loading dose before transitioning to the same 28 units/kg/h rate, with a goal heparin anti-Xa of 0.35–0.75 units/mL. Aspirin (5–10 mg/kg/day) was prescribed per provider discretion and was primarily used if there were concerns around coronary implantation during TGA repair. For Guo et al., the patients were started on aspirin at 3–5 mg/kg/day at 24 h postoperatively for septal defect repair (63).

### Long-Term Management

Long term anticoagulation therapy management was described in 60% of the selected studies. In Guo et al., participants only received aspirin and continued their postoperative antiplatelet therapy for 3—6 months, long-term dosing past six months was not reported (63). Hashmi et al., which reviewed the courses of patients undergoing cardiopulmonary bypass with acquired von Willebrand syndrome (avWS), the cohort was broadly reported as having had 44% on warfarin and 13% on aspirin after bypass (65). Sfyridis et al. documented that 41% of ToF patients were maintained on warfarin postoperatively with an INR goal 2—3 for 3 months (26). The other three manuscripts did not give details on long-term treatment (28, 35, 64). None of the studies included DOAC, DTI, or non-aspirin antiplatelet therapy.

### Monitoring

Monitoring was not reported except for the perioperative management described earlier and the INR goal of 2–3 in Sfyridis et al. as described.

### Complications

Bleeding complications were more common than thromboembolic events in this mixed group of CHD defects. Bleeding incidence ranged from 0.9% with two events, both hemothoraces, after septal defect repair (63) to 85.7% (65) in the study on patients with avWS, though no details were reported in terms of severity in the latter. Hashmi et al. concluded that the rate of bleeding is likely due to avWS and a high percentage of patients prescribed warfarin (65). In Leijser et al., they did not see any difference in postoperative stroke in TGA regardless of whether the infant was on anticoagulation (35). The use or withholding of anticoagulation did not significantly affect the incidence of subdural or intraventricular hemorrhage. The other studies in this group did not report thrombotic events clearly linked to the CHD lesions in this section.

### Summary

This group of manuscripts was more variable and heterogeneous in outcomes due to the study population and small number of included studies. Heparin infusions were commonly used initially and then transitioned to aspirin for several months postoperatively, although both enoxaparin and warfarin use was also reported. Bleeding was the most reported complication, though this range was quite broad and includes the caveat that one study was specifically evaluating an acquired bleeding disorder. No thromboembolic complications occurred and no DOACs or DTIs were prescribed.

## Conclusion

Following review of the literature published on pediatric CHD postoperative anticoagulation in the last decade, we conclude that limited controlled or prospective studies have led to widely variable management strategies. Moreover, there are very few publications highlighting the use of new anticoagulants (e.g., DOACs) that can require less monitoring than traditional therapies such as warfarin. Further research and funding in the development of novel drugs that precisely target platelets or expanding the use of newer generations of P2Y_12_ antagonists may offer alternatives to aspirin. Recent trials of antibodies and antisense oligonucleotides that block Factors XI and XII or their zymogens have shown promise in decreasing deep vein thrombosis in patients with total knee replacement since these factors play a more important role in thrombosis compared to hemostasis (66). More rigorous exploration of these therapies and standardizing older therapies may help to create consensus in future guidelines. As mortality falls for CHD surgical repair, we must focus on all phases of postoperative anticoagulation to prevent life-threatening bleeding and clotting complications.
